# CLINICAL AND EPIDEMIOLOGIC EVALUATION OF DESMOID TUMORS IN A BRAZILIAN SARCOMA REFERENCE CENTER

**DOI:** 10.1590/1413-785220243202e274225

**Published:** 2024-06-24

**Authors:** Cassia da Silva, Fábio Fernando Eloi Pinto, Ademar Lopes, Suely Akiko Nakagawa, Marcelo Porfirio Sunagua Aruquipa, Samuel Aguiar, Celso Abdon Lopes de Mello

**Affiliations:** 1 A.C. Camargo Cancer Center, Sarcoma and Bone Tumor Reference Center, Department of Surgical Oncology, Sao Paulo, SP, Brazil.; 2 A.C. Camargo Cancer Center, Sarcoma and Bone Tumor Reference Center, Departament of Oncologic Orthopedics, Sao Paulo, SP, Brazil.; 3 A.C. Camargo Cancer Center, Sarcoma and Bone Tumor Reference Center, Department of Clinical Oncology, Sao Paulo, SP, Brazil.

**Keywords:** Desmoid, Fibromatosis, Epidemiology, Desmoide, Fibromatose, Epidemiologia

## Abstract

**Introduction:**

Desmoid Tumors (DT) are rare neoplasms with higher incidence in younger women.

**Methods:**

Retrospective, single-center analysis of patients with DT. Variables were age, sex, biopsy, treatment and recurrence. The disease-free survival (DFS) was calculated with the Kaplan-Meier method.

**Results:**

242 patients were evaluated, mean age was 34 years, 70.7% women, 44.4% originated in the trunk/abdomen and 54.5% had size > 5cm. Surgery was performed in 70.2%, 31% with negative margin and only 57% with previous biopsy. Recurrence rate was 38% and 1,2,5-year DFS was 75.3%, 64.2%, 57.8%, respectively. Size (p = 0.018) and tumor location in the dorsum (p = 0.001), extremities (p = 0.003) and pelvis (p = 0.003) were related to higher relapse rate.

**Conclusion:**

our data reinforces the need to gather data from real world practice and the importance of awareness of DT and medical education about DT behavior and best approach due to the high rates of surgery and elevated number of patients treated without biopsy. ***Level of Evidence III; Retrospective Comparative Study.***

## RESUMO

**Introdução:**

Os tumores desmóides (TD) são neoplasias raras com maior incidência em mulheres jovens.

**Métodos:**

Trata-se de uma análise retrospectiva, em um único centro, de pacientes com TD. As variáveis foram idade, sexo, biópsia, tratamento e recorrência. A sobrevida livre de doença (SLD) foi calculada pelo método de Kaplan-Meier.

**Resultados:**

Foram avaliados 242 pacientes, com idade média de 34 anos, 70,7% mulheres, 44,4% com origem no tronco/abdômen e 54,5% com tamanho > 5 cm. A cirurgia foi realizada em 70,2%, 31% com margem negativa e apenas 57% com biópsia prévia. A taxa de recorrência foi de 38% e a SLD de 1, 2 e 5 anos foi de 75,3%, 64,2% e 57,8%, respectivamente. O tamanho (p = 0,018) e a localização do tumor no dorso (p = 0,001), nas extremidades (p = 0,003) e na pelve (p = 0,003) foram relacionados a uma maior taxa de recidiva.

**Conclusão:**

Nossos dados reforçam a necessidade de coletar dados da prática do cenário real e a importância da conscientização da TD e da educação médica sobre o comportamento da TD e a melhor abordagem, devido às altas taxas de cirurgia e ao elevado número de pacientes tratados sem biópsia. ***Nível de Evidência III; Estudo Comparativo Retrospectivo.***

## INTRODUCTION

Desmoid Tumors (DT), also known as Aggressive Fibromatosis, are rare neoplasms originating in connective tissues and are characterized by local deep infiltration capacity, but without metastatic potential. The most frequent location is the trunk wall and limbs but can arise in any part of the body.^1^ History of Familial Adenomatous Polyposis (FAP) syndrome is the most known risk factor and pregnancy is linked to the occurrence of abdominal wall tumors.^2^ The diagnosis is based on histomorphology with proliferation of uniform fibroblasts in a collagenous stroma with nuclear staining for beta-catenin protein on immunohistochemistry.^3^

Treatment for DT is challenging and requires a discussion at a multidisciplinary tumor board.^4,5^ When indicated, the goal of treatment is to obtain local control with the minimum possible morbidity, considering tumor location size, growing rate and patient preferences. Surgery was the main modality employed for most of the patients. However, due to the elevated rates of local recurrence after tumor resection a more conservative approach in currently advocated by most of the guidelines.^4,6^ Active surveillance is the most appropriate approach for most of patients with asymptomatic disease.

Over the last decade many trials showed promising results with systemic treatment. Conventional chemotherapy with liposomal doxorubicin, vinblastin and methotrexate are the most frequently used cytotoxic agents.^4^ More recently, tyrosine kinase inhibitors such as sorafenib^7^ and pazopanib^8^ were evaluated in prospective trials and showed response rate around 40% and good symptom control. In the prospective randomized trial that evaluated the efficacy of sorafenib, patients randomized to placebo arm presented a spontaneous tumor regression rate of almost 20%. The arsenal of systemic agents is increasing and recently, a novel agent Nirogacestat, a gamma secretase inhibitor, was evaluated in a phase III trial and proved to be effective and with adequate safety profile.

In Brazil there is limited information regarding the epidemiology of patients diagnosed with soft tissue tumors, including desmoid fibromatosis.^9^ Moreover, there is limited information regarding the clinical presentation and treatment patterns and outcomes of patients treated in Brazilian centers.^10^ The disparities in treatment access among patients in developing countries is well known and it may be more prominent in patients diagnosed with rare cancers and sarcomas as demonstrated by a large cancer database study conducted in Brazil.^9^

As a result, it is important to analyze the clinical and epidemiological aspects of patients with desmoid tumor to better guide future health policies. Our study aimed to provide real world dada of patients diagnosed with desmoid fibromatosis and treated at a large cancer center in Brazil by analyzing the clinical and demographic characteristics and to identify potential prognostic factors related with tumor relapse.

## MATERIAL AND METHODS

### Patients and variables

This is an observational, retrospective, transversal and single center study that evaluated patients treated from 1992 to 2022. Data were extracted from medical records and inserted in the Redcap platform. The project was approved by the Institutional Ethics Committee (number). The inclusion criteria were patients with diagnosis of desmoid fibromatosis, at least one treatment at the Institution, available medical data, follow up > 12 months. Exclusion criteria were concomitant active neoplasm at diagnosis. Initially 290 patients were identified, 48 were excluded due to incomplete medical information and 242 were included in the analysis.

The analyzed variables were age, sex, history of familial adenomatous polyposis, symptoms at diagnosis, history of previous local trauma, biopsy prior to treatment, type of treatment upon admission, tumor site and size, treatment received (surgery, systemic, radiation, other), status of surgical margins, disease relapse.

### Statistical Analysis

The database was constructed in the RedCap platform. Descriptive data as frequencies was presented in absolute (n) and relative (%) frequency, mean and standard deviation. To evaluate the association among qualitative variables we used the qui-squared test or the Fisher exact test and for the quantitative variables we used the t test for independent samples or the non-parametric Mann-Whitney test. The primary endpoint was Disease-Free Survival (DFS). The DFS was defined from the time of surgery to first recurrence and was estimated by the Kaplan-Meier method and the log rank-rank test was used to compare the survival curves. A *p* value <0,05 adopted in order to establish the statistical significance. SPSS version 28 was used for statistical analysis.

## RESULTS

### Population characteristics

A total of 242 patients were include and analyzed. The mean age was 34 years (1-82), 71% was female, 74% had private health insurance, 6% had history of FAP. Initial symptoms were growing mass in 52% of patients and pain in 38% and only 17% reported history of previous trauma, table 1. Primary tumor site was 44.6% in trunk and abdomen, 20.6% in extremities, 8.7 in head and neck, 25% others. Tumor size was < 5 cm in 30%, >5 and < 10 cm in 34% and > 10 cm in 18%. Biopsy had been performed in 57% of patients prior to the treatment. (Table 1)

**Table 1:** Demographic and clinical characteristics (n = 242 patients). São Paulo, 2023.

Variable	Category	N (242)	(%)
Sex	Female	171	70.7
	Male	71	29.3
Age	Mean	34	(1-82)
	< 19 years	35	14.5
	20 - 39 years	96	39.7
	40 - 59 years	53	21.9
	>60 years	13	5.4
	Unknown	45	18.5
Family history of neoplasm	Yes	126	52.1
	No	66	27.3
	Unknown	50	20.6
FAP Syndrome	Yes	14	5.8
	No	228	94.2
Initial Symptoms	Asymptomatic nodule	2	0.8
	Growing lump	145	59.9
	Pain	69	28.5
	Functional restriction	3	1.2
	Imaging finding	10	4.1
	Others	39	16.1
	Unknown	31	12.8
Previous Trauma	Yes	13	5.4
	No	201	83.1
	Unknown	28	11.5
Health plan	Public health care	46	19.0
	Health Insurance	179	74.0
	Privated (Out of pocket)	17	7.0
Location	Abdomen	91	37.6
	Head and neck	23	9.5
	Dorsum	12	5.0
	Extremities	62	25.6
	Pelvis	22	9.1
	Trunk	32	13.2
Primary tumor size	< 5 cm	83	34.3
	5-10cm	74	30.6
	>10 cm	43	17.8
	Uknown	42	17.3
Biopsy	No	61	25.2
	Yes	138	57.0
	Uknown	43	17.8

### Treatment

Upon admission only 16.9% had previous surgery and 73.6% had intact tumor and information was not available in 9.5%. After diagnosis, the initial therapeutic approach was surgery in 170 patients (70%), systemic anti-neoplastic treatment in 9.9%, radiation therapy in 2.9% and follow-up was adopted in 9.5%. (Table 2)

**Table 2:** Distribution of treatment modalites (n = 242). São Paulo 2023.

Variable	Category	N	(%)
Initial approach	observation	23	9.5
	Surgery	170	70.2
	Chemotherapy	24	9.9
	Radiotherapy	7	2.9
	others	18	7.4
	Total	242	100.0
Surgical margins	Not applicable	02	1.2
	negative	68	40.0
	positive	56	32.9
	unknown	44	25.9
	Total	170	100.0
Relapse	No	105	61.8
	Yes	65	38.2
	Total	170	100.0
Site First Relapse	Abdomen	15	23.1
	Head and neck	06	9.2
	Trunck	12	18.4
	Extremities	19	29.2
	Pelvis	13	20.0
	Total	65	100.0
Treatment first relapse	Surgery	42	55.3
	Radiotherapy	8	10.5
	Chemotherapy	12	15.8
	tamoxifen	10	13.1
	Observation	4	5.3
	Total	76	100.0
Treatment second relapse	Surgery	19	52.8
	Radiotherapy	5	13.9
	Chemotherapy	3	8.3
	Anti-inflamatory	3	8.3
	tamoxifen	5	13.9
	observation	1	2.8
	Total	36	100.0

**Figure 1:**
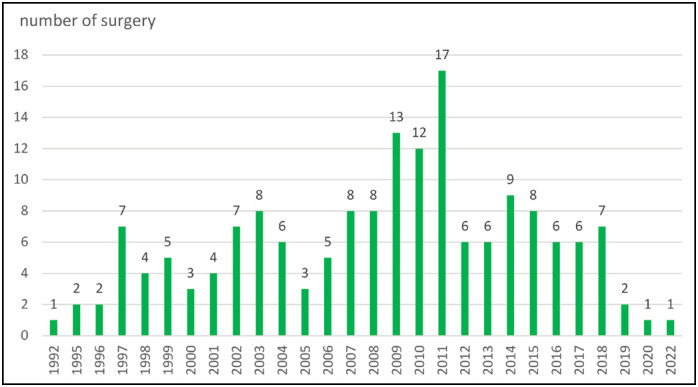
Distribution of surgical procedures to treat desmoid tumor over the years (1992-2022).

We observed a recent decrease in surgery in the past 4 years as described in Figure 1. Surgery was more frequently employed between the periods of 1992-2001 and 2002-2012 and decreased the last 3 years (2018-2022). The surgical margins status in 170/242 patients treated with surgery was negative in 40,0%, positive in 32,9% and unknown in 25,9%. (Table 2)

After first, second and third relapse, surgery was employed in 47/65, 19/30 and 7/15 of patients. (Table 2)

### Disease Free survival

**Figure 2:**
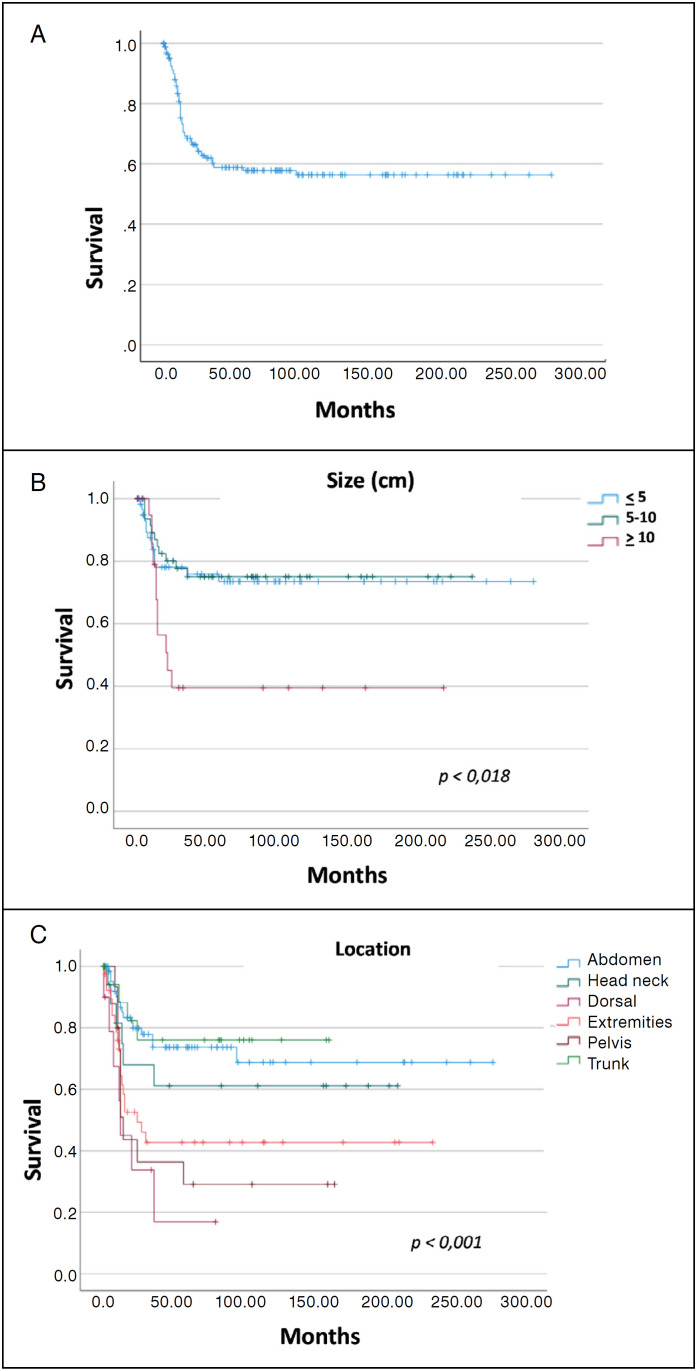
Disease-free Survival in months for patients treated with surgery (A), according to tumor size (B) and tumor location (C). Kaplan-Meier curves, log-rank test.

The median follow-up time was 91.2 months, and 65 disease relapses were observed in the group of patients treated with surgery. Health insurance (*p* = 0.001) and tumor size (*p* = 0.024) and location (*p* = 0.001) correlated with relapse (Table 3). The median DFS was not reached and the 1-, 2-, 5- and 10-year DFS were 75,3%, 64,2%, 57,8% and 56,4%, respectively. (Figure 2) Patients with tumor located in the extremities, pelvis and dorsal had inferior DFS as compared to trunk, abdomen and head and neck (24, 14, 12 months versus NR, *p* = 0,001). The median DFS for patients with larger tumors (> 10 cm) was 21 months versus NR for patients with < 10 cm tumors (*p* = 0,018). In the Cox regression analysis, patients with tumor > 10 cm had 2.5 increase in the risk of relapse (HR 2.52, CI 95% 1.14-5.59, *p* = 0.022) and tumor located in the dorsum (HR 4.69, CI 95% 1.92-11.43, *p* = 0.001), extremities (HR 2.67, CI 95% 1.32-5.16, *p* = 0.003) and pelvis (HR 3.29, CI 95% 1.49-7.29, *p* = 0.003) increased risk of relapse as compared to the other tumor locations, as shown in Table 4.

**Table 3:** Correlation of clinical variables with tumor relapse for patients treated with surgery (n = 170). São Paulo, 2023.

Variable	Relapse			^p^
	No	Yes	Total	
**Age** *				
<19	15	12	27	0.324
20-39	40	26	66	
40-59	24	9	33	
>60	6	1	7	
Total	85	48	133	
**Sex** **				
Male	33	21	54	0.868
Female	74	42	116	
Total	107	63	170	
**Health Care System** ***				
Public health care	25	6	31	0.001
Health Insurance	80	47	127	
Privaste (Out of pocket)	2	10	12	
Total	107	63	170	
**Signs and symptoms** **				
No pain	75	48	123	0.496
Pain	32	15	47	
Total	107	63	170	
**Previous trauma** ****				
No	89	49	138	0.701
Yes	4	3	7	
Total	93	52	145	
**Site** ***				
Abdomen	48	16	64	0.001
Head and Neck	12	6	18	
Dorsum	3	7	10	
Extremities	21	20	41	
Pelvis	5	10	15	
Trunk	18	4	22	
Total	107	63	170	
**Size** ***				
< 5 cm	44	11	55	0.024
5 - 10 cm	37	11	48	
> 10 cm	13	12	25	
Total	94	34	128	
**Biopsy** **				
No	42	13	55	0.687
Yes	53	21	74	
Total	95	34	129	

* Fisher-Freeman-Halton exact test;

** Continuity Correction;

*** Pearson chi-square test;

**** Fisher Exact Test.

O evaluate the association among qualitative variables we used the qui-squared test or the Fisher exact test and for the quantitative variables we used the t test for independent samples or the non-parametric Mann-Whitney test.

**Table 4:** Correlation of age, sex, tumor site and size with Disease-free survival. Cox regression model. São Paulo, 2023.

Variable	Category	HR	CI 95%		^p^
		Ref. (1)	Lower	Upper	
Age	<19	1			
	20-40	0.686	0.346	1.361	0.281
	>40	0.575	0.248	1.332	0.197
Sex	Female	1			
	Male	1.124	0.666	1.899	0.661
Site	Abdomen	1			
	Head and Neck	1.543	0.603	3.945	0.366
	Dorsum	4.693	1.926	11.438	0.001
	Extremities	2.671	1.382	5.162	0.003
	Pelvis	3.299	1.493	7.291	0.003
	Trunk	0.840	0.281	2.514	0.755
Size	< 5 cm	1			
	5 - 10 cm	0.888	0.403	1.957	0.769
	> 10 cm	2.528	1.142	5.595	0.022

Cox regression model.

## DISCUSSION

The management of desmoid tumor is challenging and the therapeutic plan should be defined by a multidisciplinary team.^4^ There are many barriers to deliver the best treatment for patients including physicians’ awareness of tumor behavior. As a result, it is important to understand the socio-demographic characteristics of patients diagnosed with DT and to evaluate the patterns of diagnosis and treatment delivered. Our study characterized the clinical and sociodemographic aspects of 272 patients with DT, the largest series of DT treated at a Brazilian cancer center.

DTs are rare mesenchymal neoplasms with uncertain behavior. Patients can present with fast growing and symptomatic tumors, or the disease can remain stable for a long time.^6^ Interestingly, some patients undergo spontaneous regression even without active treatment.^7^ Thus, an active surveillance strategy is recommended for most newly diagnosed patients.^4,6^ Our study showed that 70% of the patients underwent surgical tumor resection. This number appears to be elevated but, in accordance with the most recent guidelines,^4^ we observed a trend in the decreasing number of surgeries over the years. After surgical resection, the disease recurrence is frequent. We observed a relapse rate of 38% and the 1,2,5 years disease-free survival rate was 75,3%, 64,2%, 57,8% respectively. Our data are in line with the literature showing that most of the events occurs in the first 2 years after surgery^6^ and it could guide the follow up police after a tumor resection with more frequent medical visits and imaging in the first 2 years after surgery. There are no clear data regarding the best option for patients with tumor recurrence. The joint global consensus-based guideline focuses mainly on first diagnosis and reinforces the importance of active surveillance for most patients with asymptomatic and slow growing tumors.^4^ In our study, a salvage surgery was performed in 42/76, 19/36 and 7/17 of patients with first, second and third relapse, respectively. On the other hand, only 10% of the patients received radiation therapy and tamoxifen and 15% received systemic therapy. There are many reasons to consider a non-surgical approach after a tumor relapse. First, there is a hypothesis that growth factors released after surgery, during the initial phase of wound healing, could transmit signals that promote the activation of β-catenin resulting in tumor growth.^11^ Second, more recently prospective trials showed the activity of tyrosine kinase inhibitors to treat DT with objective response rate around 30 to 40% for pazopanib^7^ and sorafenib,^8^ respectively. Another important prospective, phase 3 trial, the DeFi trial^12^ showed that the gamma secretase inhibitor Nirogacestat promoted tumor shrinkage in almost all the patients with objective response rate of 40% and the study demonstrated an improvement in the quality of life of patients treated with Nirogacestat. Disparity and inequity in treatment access is an important barrier that patients with cancer face,^13^ especially in the Brazilian health system where there is no officially approved chemotherapy or target agent for DT. This data could partially explain the high frequency of surgery and less indication of systemic treatment. However, one the data of active systemic treatment was available only in recent years, a more detailed analysis should be carried out regarding the use of systemic treatment including tyrosine kinase inhibitors over the past 5 years.

Over the past decades, many prognostic factors, such as age, tumor size, tumor location, and surgical margins, have been associated with recurrence after surgical resection.^14,15^ Our series showed that tumor location and size were the only 2 variables associated with inferior DFS. Patients with tumors larger than 10 cm had a median DFS of 21 months as compared to NR for < 10 cm (*p* = 0.018) and tumors located in the dorsum, pelvis and limbs had median DFS of 12, 14 and 24 months, respectively and not reached in the head and neck, trunk and abdomen (p < 0,001). In the multivariate analysis, tumor size and location were independent prognostic factors related to risk of relapse. The addition of molecular profiling of DT with inclusion of CTNNB1 gene mutation status improves the accuracy of the predictive models of recurrence as demonstrated by our group in a recent study (data not published) and other authors.^16^ Moreover, the better understanding of molecular factors related to disease behavior could predict tumor progression and better guide the therapeutic approach. More recently, circulating tumor DNA (ctDNA)^17^ and circulating tumor cells^18^ are under investigation and could become a valid biomarker of response/progression.

One important step in the management of patients with soft tissue tumors is the histopathological diagnosis. The strategy of "first treat and then diagnose" is not advised since the treatment plan can only be established based on the specific tumor subtype.^5,19^ In our series, we observed a high number of patients (25%) that were treated without a previous biopsy. It may denote that patients were treated in non-reference centers for sarcomas and the suspicion of a benign lesion was made. Our data showed that growing mass and pain was present in 59% and 28% of the patients, respectively, both symptoms are not characteristics of a benign lesion. This data highlights the importance of medical education regarding initial approach of soft tissue mass and the importance of organized referral networks in the management of desmoid tumor and other sarcomas.^20^

There are some limitations in our study. First, it is a retrospective study and much information could not be retrieved from the medical records and a considerable ratio of missing information was detected. Second, important information regarding the use of hormotherapy and pregnancy was not available for most of patients. Our analysis could not detect the amputation ratio since many patients were treated with many surgical resections as well as the precise indication of surgery for recurrence. Of note, the analysis was carried out in a long period it may negatively impact the findings, but it may, on the other hand, be useful to illustrate the changes in the treatment over time. These limitations were mitigated by the large number of patients if we consider a single center analysis and for the long period of follow up.

## CONCLUSION

Our study shows the characteristics of 240 patients with the diagnosis of desmoid tumor treated at a Brazilian center. Despite the high rate of surgery, we observed a decline in the recent years. In contrast to the good practice recommendations a large proportion of patients were treated without previous biopsy. Tumor size and location were correlated with the risk of disease relapse. Our data illustrate the scenario of DT approach in Brazil and could be helpful to guide future actions in the health police strategies.
